# Barriers and Facilitators to Accessing PrEP and Other Sexual Health Services Among Immigrant Latino Men Who Have Sex with Men in Los Angeles County

**DOI:** 10.1007/s10508-024-02928-z

**Published:** 2024-07-08

**Authors:** Ronald A. Brooks, Omar Nieto, Elena Rosenberg-Carlson, Katherine Morales, Dilara K. Üsküp, Martin Santillan, Zurisadai Inzunza

**Affiliations:** 1 Department of Family Medicine, University of California, Los Angeles, 10880 Wilshire Blvd., Suite 1800, Los Angeles, CA 90024 USA; 2 Center for HIV Identification, Prevention, and Treatment Services, University of California, Los Angeles, Los Angeles, CA USA; 3 Department of Research and Evaluation, Bienestar Human Services, Los Angeles, CA USA; 4 Department of Community Health Sciences, Fielding School of Public Health, University of California, Los Angeles, Los Angeles, CA USA; 5 Department of Internal Medicine, Charles R. Drew University of Medicine and Science, Los Angeles, CA USA; 6 UCLA-CDU Center for AIDS Research, Los Angeles, CA USA

**Keywords:** HIV pre-exposure prophylaxis, Immigrant, Latino, Men who have sex with men, Sexual health services, Sexual orientation

## Abstract

In the United States, immigrant Latino men who have sex with men (ILMSM) are, compared to white MSM, disproportionately burdened by HIV and lack access to highly effective HIV prevention strategies, such as pre-exposure prophylaxis (PrEP). Qualitative research centered on exploring barriers that ILMSM experience in accessing PrEP and other sexual services is extremely limited, despite a high prevalence of HIV in this population. In this study, a purposive sample of ILMSM (*n* = 25) was recruited to participate in a semi-structured in-depth interview to identify the distinct barriers and facilitators ILMSM experience in accessing sexual health services given their complex intersectional identities of being an immigrant, Latino, and a sexual minority man. Using a thematic analysis approach, nine themes were generated from the data representing barriers and facilitators. Barriers included: (1) cost and a lack of health insurance, (2) complexity of PrEP assistance programs; (3) challenges related to the immigrant experience; (4) impact of gay stigma; and (5) communication challenges. Facilitators included: (1) improving affordability and accessibility of PrEP services; (2) receiving services from LGBT- or Latine LGBT-centered clinics; (3) receiving services from medical providers who are gay and/or Latino; and (4) providing targeted community outreach, education, and promotion of PrEP to ILMSM. While many of the barriers illuminated in the study were structural (e.g., cost and lack of health insurance), and not easy to overcome, the findings highlight a range of facilitators that can support access to PrEP and other sexual health services for ILMSM. Considering these findings, we suggest strategies that may enhance access to needed sexual health services among ILMSM.

## Introduction

In the United States, Latino men who have sex with men (LMSM) are disproportionately burdened by HIV infections. The Centers for Disease Control and Prevention (CDC) estimates that 1 in 5 LMSM will contract HIV in their lifetime compared with 1 in 11 White MSM (Centers for Disease Control and Prevention [CDC], 2021). Among Latino males, immigrant Latino MSM (ILMSM) comprise a larger percentage of new HIV infections than U.S.-born LMSM (CDC, 2023). Moreover, throughout the HIV epidemic, the proportion of new HIV diagnoses among ILMSM has continued to increase. In 2012, CDC HIV surveillance data revealed that ILMSM represented 55% of all new HIV infections among Latino males compared to 45% among U.S.-born LMSM (CDC, 2014). By 2021, CDC HIV surveillance data showed that ILMSM now comprised 69% of all new HIV infections among Latino males compared with 31% for U.S.-born LMSM (CDC, 2023). Given these trends, greater efforts are needed to increase accessibility to highly effective HIV prevention strategies, such as pre-exposure prophylaxis (PrEP), for this population.

First approved by the U.S. Food and Drug Administration in 2012 (U.S. Food & Drug Administration, 2012), PrEP has dramatically enhanced our nation’s HIV prevention efforts and is a key strategy of the federal Ending the HIV Epidemic (EHE) initiative (CDC, 2023). Unfortunately, disparities persist in PrEP uptake, particularly among minoritized populations. According to 2021 CDC data, PrEP prescriptions were highest among White populations (78%) and lowest for Latino (21%) and Black (11%) populations with an indication for PrEP (CDC, 2023). In other research, PrEP use in LMSM was only 4.3%-6.5% compared with 13.9%-22.9% in White MSM (Holloway et al., 2017; Snowden et al., 2017). There are also lower rates of PrEP use among ILMSM. In one study of LMSM in Los Angeles, California, only 5.4% of study participants were using PrEP and the majority (81%) of PrEP users were U.S.-born (Brooks et al., 2019). The persistent low rates of PrEP uptake are likely to perpetuate high rates of HIV infections among ILMSM.

Several studies have found that LMSM experience multiple barriers to accessing PrEP and other sexual health services. These include structural factors such as cost of HIV prevention services, lack of access to healthcare, and difficulty navigating complex healthcare systems (Harkness et al., 2023; Page et al., 2017; Tieosapjaroen et al., 2023). Other barriers to PrEP access include medical mistrust, PrEP stigma, experiences of discrimination, and discomfort communicating with medical providers about sexual behaviors (Harkness et al., 2023; Lelutiu-Weinberger & Golub, 2016; Lozano et al., 2023). In some of this prior work, ILMSM are included as part of the study population and their unique barriers to accessing services have been identified (i.e., language issues, immigration concerns) (Lozano et al., 2023). However, ILMSM were not the explicit focus of these studies, and most have been limited to the southern and eastern regions of the U.S. (Cantos et al., 2023; Harkness et al., 2023; Lelutiu-Weinberger & Golub, 2016; Lozano et al., 2023). Furthermore, most of these studies are quantitative in nature and use surveys with standardized assessments, thus excluding the opportunity for a more in-depth qualitative exploration of the experiences of ILMSM. Additionally, few studies have explored experienced stigma and/or have examined intersectional stigma as it relates to accessing PrEP services. Qualitative research that is focused on examining impediments that ILMSM experience in accessing sexual health services is sparse, despite this population being heavily burdened by HIV.

In the U.S., ILMSM live at the intersection of multiple socially disadvantaged positions (i.e., being an immigrant, Latino, and a sexual minority man), which may impede their access to needed HIV prevention services (Berger, 2010; Bowleg et al., 2013; Crenshaw, 1989; Dale et al., 2022; Dworkin, 2005). Given existing health inequities, structural racism, and discrimination, these disadvantaged positions can render fewer resources and less access to healthcare services for ILMSM (Misra et al., 2021; Philbin et al., 2018; Viruell-Fuentes et al., 2012; Watkins-Hayes, 2014). As part of HIV prevention efforts, it is important to be mindful of the complex identities and unique barriers to care that ILMSM experience when designing HIV prevention services for this marginalized population.

Access to healthcare is seen as a complex concept that must be evaluated from multiple positions (Gulliford et al., 2002). Having available services, though critical, does not guarantee service utilization. Utilization may be limited by financial, organizational, social, or cultural barriers. Therefore, as the focus of the present study, we sought to identify barriers and facilitators to accessing PrEP and other sexual health services experienced by ILMSM in Los Angeles County (LAC). Addressing issues of access is critical to bringing an end to the HIV epidemic, particularly in a HIV epicenter like LAC where the population most affected by HIV are Latino males, who in 2022 represented the largest percentage (41%) of all persons living with a HIV diagnosis (Division of HIV and STD Programs, Los Angeles County Department of Public Health, 2022).

Based on 2022 census data, people of Latino/Hispanic origin comprised the largest ethnic/racial group (49%) in LAC (Los Angeles Almanac, 2024a). Furthermore, 40% of Latino/Hispanics in LAC are immigrants, 38% Spanish speakers, and an estimated 13% undocumented (Los Angeles Almanac, 2024a; USC Dornsife Equity Research Institute, 2022). To provide protections for immigrant populations, LAC has adopted “sanctuary” policies prohibiting any public employee, including law enforcement officers, from being utilized for federal immigration enforcement (Los Angeles Almanac, 2024b), which allow undocumented ILMSM to access sexual and other healthcare services without fear of legal repercussions. Additionally, in 2024, California became the first state in the nation to offer Medicaid benefits (i.e., Medi-Cal) to all eligible undocumented immigrants after expanding coverage to undocumented immigrants through age 25 in 2019 and over age 50 in 2022 (Western Center on Law & Poverty, 2024). These policies and the makeup of LAC’s large Latino population make LAC an ideal setting for conducting the current study with ILMSM.

## Method

### Participants

Between February and July 2022, a purposive sample of ILMSM was recruited to participate in a semi-structured in-depth interview. Potential participants were recruited through community-based organizations, social media postings, and participant referrals, and then screened by phone to determine eligibility. To be eligible, participants had to be at least 18 years of age, foreign-born, Latino/Hispanic ethnicity, gay/bisexual/MSM, had to have accessed or attempted to access sexual health services including PrEP, and had to reside in LAC. Recruitment was terminated once data saturation was reached (Hennink et al., 2017).

### Measures and Procedure

An interview guide was created to explore ILMSM’s experiences accessing sexual health services given their socially marginalized identities of being an immigrant, Latino, and gay/bisexual/MSM, with some questions adapted from prior research on intersectional stigma and PrEP use among Black MSM (Quinn et al., 2019). As part of the interview, participants were asked to consider these three identities when describing: (1) experiences accessing PrEP, HIV testing, and STI screening and treatment services; (2) barriers they encountered when accessing these services; (3) the extent to which personal identities such as being an immigrant, Latino, sexual minority man, and/or a monolingual Spanish speaker impacted their ability to access services; (4) the impact of PrEP stigma (i.e., the negative views or opinions people have about individuals who use PrEP) on their experiences accessing services; and (5) what they need from healthcare facilities (i.e., existing and potential facilitators) to improve access to PrEP. Interviews were conducted in both English and Spanish over Zoom, were audio recorded, and lasted 30–60 min. A demographic survey was administered through Qualtrics.

Written informed consent was obtained from all participants prior to the interview, and participants received a $50 e-gift card in compensation. Each participant was assigned a unique participant identification number to maintain confidentiality. All interviews were transcribed verbatim and Spanish-language interviews were translated into English.

### Data Analysis

The study team used a thematic analysis approach to analyze the data (Braun & Clarke, 2006, 2012). A codebook was developed through an iterative process, which began with the study team familiarizing themselves with the transcripts and noting any preliminary codes and themes. The resulting coding scheme consisted of both deductive codes from the interview guide and field notes, and inductive codes from the review of transcripts. Study team members RAB, ON, and KM then met weekly to test the preliminary codebook with multiple transcripts (*n* = 7). As part of this process, further clarifications, modifications, conditional code criteria, and refinement were made to each code, and intercoder discrepancies were discussed by the full team and resolved before final consensus was reached on the codebook. Once finalized, intercoder agreement (ON and KM) was calculated by randomly selecting and coding 20% (*n* = 5) of all interview transcripts. The Cohen’s kappa value was calculated to be *k* = 0.86, indicating a high level of agreement before final coding occurred (McHugh, 2012; O’Connor & Joffe, 2020). Team members ON and KM then coded all remaining transcripts in Dedoose (version 9.0.54).

To construct themes, the team reviewed and clustered coded data that were similar and represented a meaningful pattern in relation to the focus of the study on identifying barriers and facilitators to accessing PrEP and other sexual health services among ILMSM in LAC. Themes were then reviewed to ensure that they represented the most important and relevant components of the data.

## Results

Twenty-five ILMSM completed the study interview, with 15 interviews conducted in Spanish and 10 conducted in English. Table 1 provides participant demographic characteristics. The mean age of participants was 24 years (range = 21–70) and the average years living in the U.S. was 18 (range = 1–44). Most participants identified as Mexican (58%), gay (88%), had some level of college education (68%), were employed (88%), and had some form of health insurance (64%). Immigration status varied: 36% were undocumented, 20% were naturalized U.S. citizens, 16% were permanent residents (i.e., green card holders), 16% were here under a temporary visa (i.e., student, tourist, or work visa/permit), and 12% had temporary protection status (i.e., asylum or refugee status).

**Table 1:** Participant demographic characteristics, knowledge of PrEP, and PrEP use characteristics (*N* = 25)

Variable	*N* (%) or Mean, Range
*Demographic characteristic*	
Age (*N* = 24)^a^	Mean = 39 Range = 21–70
*Language of interview*	
Spanish	15 (60%)
English	10 (40%)
*Latino ethnicity (N* = *24)*^*a*^	
Mexican	14 (58%)^b^
Salvadorian	3 (13%)
Brazilian	2 (8%)
Bolivian	1 (4%)
Columbian	1 (4%)
Nicaraguan	1 (4%)
Peruvian	1 (4%)
Venezuelan	1 (4%)
*Race*	
White	8 (32%)
Black or African American	3 (12%)
Bi/Multi-Racial	1 (4%)
Other Race^c^	13 (52%)
*Sexual orientation*	
Gay	22 (88%)
Bisexual	2 (8%)
Queer	1 (4%)
Years living in US	M = 18, Range = 1–44
*Current immigration status*	
Undocumented	9 (36%)
Naturalized US citizen	5 (20%)
Permanent resident (i.e., green card holder)	4 (16%)
Temporary resident (i.e., student, tourist, or work visa/permit)	4 (16%)
Temporary protect status (i.e., asylum or refugee status)	3 (12%)
*Education*	
Never attended school	1 (4%)
Less than high school	2 (8%)
High school / GED	5 (20%)
Associate degree	7 (28%)
Bachelor’s degree	8 (32%)
Graduate degree	2 (8%)
*Employment status*	
Unemployed	1 (4%)
Working full-time	15 (60%)
Working part-time	7 (28%)
Retired	1 (4%)
Other^d^	1 (4%)
*Income (N* = *23)*^*a*^	
$10,000-$19,999	6 (26%)^b^
$20,000-$39,999	11 (48%)
$40,000-$59,999	6 (26%)
*Health insurance*	
Uninsured	9 (36%)
Employer-provided insurance	5 (20%)
Medicare	3 (12%)
Medi-Cal/Medicaid	2 (8%)
Self-purchased health insurance	2 (8%)
Multiple health insurance	1 (4%)
Other^e^	3 (12%)
*Knowledge of PrEP*	
Only a little	8 (32%)
A moderate amount	10 (40%)
A great deal	4 (16%)
A lot	3 (12%)
*Ever used PrEP*	
Yes	17 (68%)
No	8 (32%)
*Currently using PrEP*	
Yes	12 (48%)
No	13 (52%)
Length of time on PrEP (in months) (*N* = 11)^f^	M = 17, Range = 0.25–36
*Interested in PrEP (N* = *9)*^*g*^	
Yes	8 (89%)
No	1 (11%)

^a^Only includes participants who responded to the question

^b^Percentages were rounded to the nearest whole number and may not add up to 100%

^c^Other race includes: “Ispano,” “Latino,” “Latinx,” “Mexican,” and “Mexico Americano.”

^d^Other employment includes: “Disabled.”

^e^Other health insurance includes: “Bienestar Human Services,” “Emergency medical care,” and “I am not sure.”

^f^Only includes current PrEP users who responded to the question

^g^Only includes non-PrEP users who responded to the question

Nine themes were generated from the data representing barriers and facilitators to accessing PrEP and other sexual health services among ILMSM. Barriers included: (1) cost and a lack of health insurance, (2) complexity of PrEP assistance programs; (3) challenges related to the immigrant experience; (4) impact of gay stigma; and (5) communication challenges. Facilitators included: (1) improving affordability and accessibility of PrEP services; (2) receiving services from LGBT- or Latine LGBT-centered clinics; (3) receiving services from medical providers who are gay and/or Latino; and (4) providing targeted community outreach, education, and promotion of PrEP to ILMSM.

## Barriers

### Cost and a Lack of Health Insurance

Cost and a lack of health insurance emerged as a prominent barrier that hindered ILMSM from accessing sexual health services. For some ILMSM, their immigration status made them ineligible to apply for health insurance and other financial assistance programs that could help offset the costs of PrEP:

Well, the fact that as an [undocumented] immigrant, I don’t qualify for assistance programs, and I don’t have the economic solvency to pay for private hospitals. I must look for free services at organizations, and that sometimes delayed my access to some services I’ve needed in the past. (Age 27, Mexican, 3 years living in the U.S., Undocumented, 24 months on PrEP)

Because they lacked insurance, ILMSM could not receive services from traditional healthcare networks and were forced to seek out other clinics where they could obtain PrEP for free or at low cost (e.g., LGBT-centered clinics). This experience was shared by one participant: “I must search among the free options…. in this case, the LGBT Center” (Age 27, Mexican, 3 years living in the U.S., undocumented, 24 months on PrEP). Even when insured, some participants were unable to access PrEP due to the high copayment and other associated costs:

In the end, I wasn’t eligible [for a free PrEP program] because I have medical insurance, but my insurance’s copayment is too expensive. (Age 50, Mexican, 30 years living in U.S., Naturalized U.S. citizen)

### Complexity of PrEP Assistance Programs

In general, ILMSM found the process of applying for PrEP assistance programs to be complex because they were required to fill out excessive amounts of paperwork or provide proof of income or employment to qualify:

They [program staff] were asking me for tax statements about how much I had made and because I hadn’t worked, well, I couldn’t give them any statements and I couldn’t go to my appointments. (Age 36, Mexican, 16 years living in the U.S., Undocumented)

Because of the complexity involved in applying for these programs, some participants opted to forgo PrEP altogether:

At least, for me, it was one of the things that discouraged me the first time, because I said, “I’m not going to fill that—” I don’t know how many forms there were. It was like 10, 20, I don’t know. I said, “Ah, I don’t need it [PrEP].” (Age 30, Venezuelan, 6 years living in U.S., Temporary protected status)

### Challenges Related to the Immigrant Experience

The immigrant experience in the U.S. posed multiple challenges for ILMSM when accessing sexual health services. For example, when first arriving to the U.S., many ILMSM lacked knowledge about where and how to access services. In addition, participants expressed that they either did not know whom to ask or were “embarrassed” to reach out for help in accessing these services:

What I would just maybe emphasize is just how difficult it was for me when moving to this country to just learn that it is okay to reach out for help when it comes to sexual health, whether it's HIV screening or STI testing or treatment… I was so embarrassed to talk to friends. I have no family here, so who can I talk to? (Age 31, Bolivian, 7 years living in the U.S., Temporary resident, 12 months on PrEP)

As persons not born in the U.S., some ILMSM reported feeling isolated (i.e., like an “*outcast*”) or excluded from HIV prevention services. More importantly, participants felt like they were not “deserving” of these public services:

[I thought] maybe I didn't deserve health treatment because I wasn't paying taxes. So, I was using a public service and yeah, that was my mind at that time when I started it [PrEP]. (Age 29, Brazilian, 6 years living in the U.S., Undocumented, 12 months on PrEP)

Additionally, ILMSM were hesitant to access services because they feared that their personal identifiable information would be reported to or shared with immigration authorities, the State government, or employers. One participant expressed, “I am worried, for example, that it could be reported to the State and tied to identifying information like my name” (Age 50, Mexican, 30 years living in the U.S., Naturalized U.S. citizen). ILMSM also feared that accessing these services would negatively impact the immigration process and lead to their visa being revoked, as indicated by the following quote:

Every time I come back [from Mexico], it’s with a lot of uncertainty because I don't know if the services I’ve used will share the information with Immigration, which will prevent me from accessing the country in the future. (Age 27, Mexican, 3 years living in the U.S., Undocumented, 24 months on PrEP)

### Impact of Gay Stigma

Gay stigma emerged as an additional factor hindering ILMSM’s access to sexual health services. Participants described instances where they experienced homophobia or discrimination in healthcare settings, which led them to discontinue services:

But the fact that I belonged to the LGBT community, I think that was really something that wasn't really welcome… So, I just felt I had to take a walk. Though, the fact remains that I was called back, but I just felt since I had left there, I didn't want to go back there. (Age 31, Mexican, 15 years living in the U.S., Permanent resident)

In some cases, the lack of acceptance participants experienced in their home country affected how they thought they would be received by staff at healthcare facilities in the U.S., which subsequently impacted their willingness to disclose sexual behaviors. This point is demonstrated in the following two quotes:

Maybe I’m sometimes the nervous [one], that they’re going to say to me, ‘You’re like that. You’re gay, right?’ […] Because the small town where I’m from in Mexico, I did have a lot of very unpleasant experiences because of the way I am, because of my sexual orientation. (Age 44, Latino ethnicity unknown, 20 years living in the U.S., Undocumented)

I definitely feel more safe regarding those topics [disclosing same-sex behavior] here in the U.S., but still my fear would remain. I still would feel hesitant to talk about those things. Because in general, I'm not sure, you have to put up this barrier too and just assume that people may not be okay with your sexuality or sexual identity. (Age 21, Columbian, 1 year living in the U.S., Temporary resident)

### Communication Challenges

ILMSM also encountered challenges communicating with staff when engaging with services. For example, some participants found it difficult to communicate their needs to staff because of their limited English language proficiency. One participant noted, “Sometimes it is a bit difficult because you can’t find a way to ask it. More than anything, to ask any concerns you have” (Age 40, Mexican, 16 years living in the U.S., Undocumented). Other participants felt that the translation services provided were inadequate or created situations where they were either uncomfortable or embarrassed discussing their sexual behaviors with multiple individuals (i.e., medical provider and interpreters). This sentiment is captured in the following quote:

You just had to talk to more people about your sexual experiences, which made it a bit more awkward… In the beginning, when I didn't speak the language, I needed an interpreter, which made it a bit more uncomfortable because I had to share information with more people. (Age 27, Mexican, 3 years living in the U.S., Undocumented, 24 months on PrEP)

## Facilitators

### Improving Affordability and Accessibility of PrEP Services

Among participants, a perceived facilitator to using PrEP is to offer PrEP services (e.g., medication, doctor’s visits, labs) for free or at a reduced cost, as captured in these two quotes:

No one should be denied the right to receive PrEP, especially when it’s medication that prevents a virus like HIV, which is also a public health issue…. I think that’s something that should be free, or at least low cost for everyone. (Age 30, Venezuelan, 6 years living in the U.S., Temporary protect status)

I don’t know if treatment is still expensive but lowering its price would help…. It would be nice if the pharmaceutical companies reduced the price, or if they helped with discounts or something to-- If the clinics offered the service, too, not for free, but at a low price to be able to access the service. (Age 55, Mexican, 30 years living in the U.S., Undocumented)

To improve accessibility, ILMSM suggested increasing the number of clinics that can provide PrEP and building the capacity of smaller, specialized LGBT-centered clinics to handle larger numbers of ILMSM patients, as indicated by the following participant:

So, if we can have more centers that people can access PrEP from, I think it's going to be a good idea […]. I think that maybe many people need treatment [PrEP] but can’t access it because the clinics lack capacity. (Age 27, Mexican, 3 years living in the U.S., undocumented, 24 months on PrEP)

### Receiving Services from LGBT- or Latine LGBT-Centered Clinics

The organizational culture of settings where ILMSM received services was an important facilitator to support access to sexual health services. ILMSM sought out LGBT or Latine LGBT-centered clinics because they were affirming, supportive, non-stigmatizing or judgmental, and culturally appropriate, which allowed participants to feel comfortable talking about their sexual behaviors. These views are captured in the following quotes:

Honestly, I never had a bad experience here [at a Latine LGBT-centered clinic]. But I guess, most of them [staff] identify themselves as queer, so I think that helps a lot. Yeah, that definitely makes me feel more comfortable and welcomed. I feel like I'm among friends and they understand me, and they respect people because they've been through the same thing. (Age 29, Brazilian, 6 years living in the U.S., Undocumented, 12 months on PrEP)

The truth is that I can speak openly [at an LGBT-centered clinic] about my experiences and the situations that have led me to that consultation with the staff. They are very respectful and are always willing to support us with a good attitude. (Age 27, Mexican, 3 years living in the U.S., Undocumented, 24 months on PrEP)

The importance of LGBT specialized clinics was further highlighted when compared to other so-called “regular” clinics or hospitals that may not be welcoming for LGBT people:

I mean, there’s a big difference when you know that you’re going to a clinic that’s related to us [LGBT community] compared to just any clinic or hospital. I mean, so it is really important that there’s a place where you know you can go with that confidence and with that assurance that they’re going to listen to you and they’re going to help you.... (Age 36, Mexican, 16 years living in the U.S., Undocumented)

However, knowing where to receive services in a safe and supportive environment was something that ILMSM learned over time after arriving in the U.S., as noted by this participant, “I think as I spent more time in the US, so I understood how things work and that I should go to the LGBT clinics so my life would be easier. I kind of learned a lesson” (Age 29, Brazilian, 6 years living in the U.S., Undocumented, 12 months on PrEP).

### Receiving Services from Medical Providers Who Are Gay and/or Latino

For ILMSM, an additional facilitator for enhancing utilization of services is to receive these services from medical providers that match one or more of their identities (i.e., Latino and/or gay). Providers with similar backgrounds were thought to have greater empathy for the lived experiences of ILMSM, made participants feel understood, and inspired a sense of confidence in the services they were receiving, as discussed by these two participants:

What makes it easier is that when I come in, I’m welcomed by a person of my own race [Latine ethnicity], and it makes it easier. It makes me more comfortable, and I feel that the doctor will understand everything. (Age 70, Mexican, 42 years living in the U.S., Naturalized U.S. citizen)

I believe that kind of person [gay provider] would understand me better than someone outside the [LGBT] community. (Age 27, Mexican, 13 years living in the U.S., Permanent resident, 23 months on PrEP)

ILMSM also felt they would be more willing to engage with services if they were seen by a Latine provider who spoke Spanish:

I would feel more comfortable because of the language if they were Latino. I don’t care if she’s gay or not, to be honest, but if they’re Latino, yes, because I feel more trust and the communication is more open… My first language is Spanish… I can communicate in English, but I would feel more comfortable if she spoke Spanish. (Age 48, Mexican, 25 years living in the U.S., Undocumented)

### Providing Targeted Community Outreach, Education, and Promotion of PrEP to Immigrant Latino Men Who Have Sex with Men

Finally, participants felt there was a lack of PrEP knowledge among ILMSM and a great need for community outreach, education, and promotion to inform ILMSM about PrEP, including where and how to access it. This view is captured in the following comment:

I think giving more information, being more open, letting the community know how to receive PrEP, if they can qualify or information about PrEP. The side effects and all that. I think we do need a little bit more information to improve the service. (Age 44, Latino ethnicity unknown, 20 years living in the U.S., Undocumented)

Participants also stressed that PrEP promotional materials need to build trust and confidence in PrEP as a safe and effective product for ILMSM:

I mean, create more trust, give more details. I mean, I know that’s the most important thing, that you’re not going to catch HIV… but they should give you more security.... Like getting rid of all those negative things that people, for example, might have. Those doubts that sometimes make you hesitant to have treatment [use PrEP]. (Age 36, Mexican, 16 years living in the U.S., Undocumented)

Additionally, participants suggested that promotional materials include information or transparency about whether non-U.S. citizens are eligible for PrEP services, as captured in this comment, “I think being as clear as possible regarding if a non-US citizen is eligible for these types of services…that would help a lot” (Age 21, Columbian, 1 year living in the U.S., Temporary resident). These promotional materials also need to be translated into Spanish to improve comprehension and access, and inspire a feeling of inclusion for non-English speaking ILMSM, as explained by this participant:

I know in a lot of cases, English may not be the main language of a lot of people…. I think [if] they [promotional materials] would be translated or [if] they would be more accessible for people whose first language was not English, then that would be great because there'd be more access. You'd have more access to those types of things. And then also you would see that those things aren't just meant for U.S. citizens. (Age 21, Columbian, 1 year living in the U.S., Temporary resident)

## Discussion

This study sought to identify barriers and facilitators to accessing sexual health services among ILMSM residing in LAC, a high HIV prevalence EHE jurisdiction. The project also sought to address a gap in the literature, given that few studies have focused on ILMSM exclusively and have used a qualitative approach to explore issues of access to sexual health services in this population with multiple intersecting marginalized identities. The lack of research on impediments to needed sexual health services among ILMSM is of concern given the trajectory of increasing HIV prevalence in this population. To get at the experiences of ILMSM, we used lead in phrases to drive participants to think about their multiple identities when answering questions (e.g., “For this next set of questions, I want you to think about your multiple identities, including being Latino, gay/bisexual, and foreign-born, when describing your experiences in accessing sexual health and HIV prevention services”). We found the barriers experienced by ILMSM, many of which are structural (e.g., cost and lack of health insurance), are difficult to overcome. Fortunately, participants highlighted multiple facilitators that can support ILMSM in accessing sexual health services. In the following paragraphs, we expand on the major barriers and facilitators identified in the study and offer recommendations for improving access to needed services among ILMSM.

Most predominant for ILMSM in this sample were the structural- and community/ organizational-level barriers they encountered when accessing services. Financial barriers were a primary concern as well as the complexity of enrolling in PrEP assistance programs, which are consistent with previously published barriers among individuals from other racially/ethnically diverse MSM populations (Dolwick Grieb et al., 2015; Harkness et al., 2023; Page et al., 2017; Rhodes et al., 2010). Fortunately, California residents who are not eligible for full PrEP coverage through an insurer can receive financial support through the State’s PrEP Assistance Program (PrEP-AP) regardless of immigration status. Local clinics and community-based organizations that serve the community can also help make ILMSM aware of this option and connect them with patient navigators to ensure they receive the financial assistance they need to access PrEP.

At the organizational-level, gay stigma served as a challenge for participants when accessing sexual health services and typically manifested as experiences of homophobia or discrimination in healthcare settings. As noted in prior studies with other MSM populations, these experiences made it difficult or uncomfortable to access services (Cahill et al., 2017; Mayer et al., 2020; Rice et al., 2019; Wells et al., 2024). To address these barriers, staff in healthcare settings—from front desk staff to healthcare providers—should receive education and training on how to provide culturally sensitive and appropriate services to MSM populations, particularly ILMSM who may experience more challenges engaging with healthcare services. Several past studies have found LGBTQ + cultural competency trainings were an effective way to improve health professionals’ attitudes and behaviors toward LGBTQ + patients (Yu et al., 2023).

An important contribution of the current study was the identification of barriers and challenges unique to ILMSM. Participants described how they felt isolated while living in the U.S. and that they did not deserve access to healthcare services because they were foreign-born, demonstrating internalized stigma of being an immigrant in the US. Several studies point to the importance of utilizing social networks as a resource to both increase uptake of HIV prevention behaviors (e.g., HIV testing) among ILMSM and mitigate the stressors of living in the U.S. as an immigrant (Barrington et al., 2018; Painter, 2018). In addition, when first arriving in the U.S., ILMSM in our study commonly reported not having any knowledge of available sexual health services important to remain HIV negative, and that this knowledge was only acquired over time. Gaining this HIV prevention knowledge can be challenging without family or support systems to rely on. In the absence of existing social support networks, ILMSM serving as peer health educators might be an optimal strategy to facilitate broader reach and cultural congruency of HIV prevention education for ILMSM (Rhodes et al., 2016).

The current study also found that immigration status was a barrier for ILMSM, particularly those with no documentation or with temporary or short-term visas. As a result, some participants feared that accessing PrEP and other sexual health services might jeopardize their ability to apply for citizenship in the future or lead to the revocation of their current visa, which is consistent with previous research (Galletly et al., 2023; Gilbert & Rhodes, 2013; Lozano et al., 2023; Rhodes et al., 2010). Fortunately, federal law now supports undocumented immigrants to receive public health benefits without threat to their pursuit of legal residency (U.S. Department of Health & Human Services, 2022). Public awareness campaigns about these legal protections may help support ILMSM and other immigrant populations to seek services without fear.

A key barrier unique to our monolingual Spanish-speaking participants was the communication challenges they experienced when accessing sexual health services (i.e., language barriers). This has also been noted in other studies with Spanish-speaking Latino populations (Garcia & Duckett, 2009; Lozano et al., 2023; Spadafino et al., 2016). For ILMSM who need translators, the communication challenges are amplified because of the sensitive nature of the conversations they are having with medical providers (e.g., discussions about same-sex behavior and other HIV risk behaviors). ILMSM can feel awkward or uncomfortable when the patient-provider communication involves a third party (i.e., interpreter), which may prevent ILMSM from discussing their sexual health needs or requesting HIV prevention information (Rhodes et al., 2010). Strategies such as hiring Spanish-speaking health professionals and ensuring relevant materials are available in Spanish can help mitigate language barriers.

Our findings further highlight some of the existing and proposed facilitators to enhance access to services among ILMSM. The presence of LGBT- or Latine LGBT-centered clinics was a central facilitator for ILMSM to access services. For participants, these community resources provided a safe and affirming space for ILMSM to receive sexual health services in a non-judgmental environment. However, because only a small number of these types of clinics exist, participants suggested a need to create more facilities like these and to improve the ability of the existing clinics to serve a larger number of ILMSM. Prior research has noted the importance of LGBT centers in providing healthcare and other social services for LGBT populations (Martos et al., 2017, 2019). Expanding these types of healthcare spaces for LGBT populations is much needed and can support greater health equity for the population.

Participants also described the benefits of receiving services from medical providers who share one or more of their identities (i.e., Latino, gay). These providers allow ILMSM to feel more comfortable talking about their same-sex behavior and sexual health needs without fear of judgment, stigma, or ridicule. Previous research suggests that providers who share the same sexual orientation as their patients might have a greater understanding of the sexual and social needs of their gay male patients (Devarajan et al., 2020). Our findings also suggest a need to expand the number of Latino providers available in healthcare settings to help facilitate access to sexual health services among ILMSM patients, particularly those who are Spanish speaking.

Additionally, participants in the current study noted a lack of PrEP awareness among ILMSM, and a lack of clarity about whether existing PrEP services are meant for them. As a result, community outreach and promotion were considered potential facilitators to increase access to and understanding of PrEP services. However, participants felt that promotional materials must be tailored to ILMSM so that they know the messaging is directed at them and addresses their unique circumstances as an ILMSM. Additionally, there is a significant need to improve the policy literacy of ILMSM so that they understand their rights as immigrants in accessing free or publicly funded sexual health services.

This study was not without limitations. The study was conducted in Los Angeles, California, which has adopted “sanctuary” policies regarding immigrants. As such, these findings may not reflect barriers ILMSM might experience in less welcoming environments without any local protections and where anti-immigrant sentiment is more prevalent. An additional limitation is that our interview questions were limited in focus and could have gone beyond the individualized identities of participants to explore barriers at multiple levels, such as social structures and inequalities. Furthermore, we could have used more open-ended questions when asking about PrEP stigma so as not to bias participants into solely understanding their experience as being impacted by PrEP stigma when accessing PrEP. A final limitation is that all interviews were conducted over Zoom and may have excluded ILMSM with limited access to and/or comfort using videoconferencing technologies.

### Conclusion

This study contributes to existing research on barriers to sexual health services experienced by MSM populations by identifying barriers unique to a population with multiple intersecting and stigmatized identities (i.e., being immigrant, Latino, gay/bisexual) (Collins et al., 2021; Crenshaw, 1989). However, not all identities of ILMSM are marginalized. Future research should explore intersecting identities and group identity characteristics that are supportive or can facilitate positive health outcomes in this population. One example is the collectivist nature or group identification of Latinos that can be protective and serve as a support system or resource for ILMSM when accessing healthcare services (Elder et al., 2009; Wells et al., 2024). Other future research directions include piloting the strategies noted above to assess their effectiveness in facilitating access to PrEP and other sexual health services among ILMSM.
